# Clozapine Withdrawal Catatonia: A Case Report

**DOI:** 10.7759/cureus.52462

**Published:** 2024-01-17

**Authors:** Amit Jagtiani, Tarika Nagi, Raghu Gandhi, Abid Rizvi

**Affiliations:** 1 Psychiatry, Burrell Behavioral Health, Springfield, USA; 2 Child and Adolescent Psychiatry, Harlem Hospital - Columbia University Medical Center, New York, USA; 3 Psychiatry, Columbia University College of Physicians and Surgeons, New York, USA; 4 Psychiatry, Abbott Northwestern Hospital, Minneapolis, USA; 5 Psychiatry and Behavioral Sciences, West Virginia University, Morgantown, USA

**Keywords:** schizoaffective, schizophrenia, catatonia, withdrawal, clozapine

## Abstract

Catatonia, which is associated with gamma-aminobutyric acid (GABA) hypoactivity, often responds robustly to benzodiazepines. It has been reported to be a consequence of abrupt discontinuation of clozapine, an antipsychotic used for treatment-resistant schizophrenia. Clozapine discontinuation, sometimes necessitated by medical concerns, can carry the risk of adverse outcomes, including catatonia. We present the case of a 66-year-old African-American male with schizoaffective disorder (depressive subtype) and a complex medical history. He discontinued clozapine abruptly due to medication unavailability, and, seven days later, presented with catatonic symptoms, initially unrecognized by emergency room clinicians. His symptoms included self-neglect, auditory hallucinations, isolation, psychomotor retardation, fixed gaze, and thought blocking. An attempt to reinstate clozapine led to orthostatic hypotension, prompting admission to an inpatient psychiatry unit. Attempt to initiate risperidone for psychosis worsened the catatonia, which then responded rapidly to intravenous lorazepam challenge. This facilitated the re-introduction of clozapine with slow re-titration.

## Introduction

Catatonia is a neuropsychiatric condition believed to be closely associated with gamma-aminobutyric acid (GABA) hypoactivity, as evidenced by its robust response to benzodiazepines [1]. While initially described in case reports primarily in the context of benzodiazepine withdrawal [2], catatonia has also been observed as a consequence of abrupt discontinuation of clozapine [1]. Clozapine is an antipsychotic often used for treatment-resistant schizophrenia. Clozapine discontinuation is frequently necessitated in clinical practice, particularly in cases of blood dyscrasia or suspected myocarditis, and sometimes occurs abruptly for various reasons. Such abrupt cessation of clozapine is recognized to carry the risk of several adverse outcomes, including rebound psychosis [1], dyskinesias [3], and, albeit rarely, the induction of catatonia [1].

## Case presentation

We present the clinical case of a 66-year-old African-American man with a diagnosis of schizoaffective disorder (depressive subtype). His medical history included chronic obstructive pulmonary disease, type 2 diabetes, vitamin D deficiency, and hypertension. The patient had been effectively managing his condition through a medication regimen consisting of 100 mg of clozapine administered orally twice daily, in conjunction with a daily dose of 20 mg of escitalopram, as prescribed by the psychiatry clinic. However, he abruptly discontinued his clozapine usage due to running out of his medications.

Notably, seven days following the cessation of clozapine, he exhibited catatonic symptoms when he presented to the emergency room (ER). These symptoms were initially overlooked by the ER clinician. The patient's complaints upon presentation included self-neglect, neglect of his elderly father, and self-reported auditory hallucinations. In the ER, he displayed isolation, psychomotor retardation, a fixed gaze at a wall, and thought blocking, and vaguely endorsed auditory hallucinations and suicidal ideation (responding with "maybe" when questioned about them). In an attempt to address his condition, he was promptly reintroduced to clozapine at a dose of 25 mg twice daily in the ER. Unfortunately, this resulted in orthostatic hypotension and a subsequent fall. Consequently, clozapine was discontinued, and he was admitted to an inpatient psychiatry unit for stabilization.

During his stay on the psychiatry floor, the patient displayed poor food and fluid intake. His treatment regimen was adjusted to include risperidone, beginning at 0.5 mg twice daily and gradually increasing to 2 mg twice daily by the 13th day of admission. Risperidone was chosen due to difficulty in re-initiating clozapine in the ER. On the 14th day, he presented with tachycardia and elevated blood pressure, prompting an urgent medical consultation. Extensive diagnostic tests, including an electrocardiogram, CT scan of the chest, and vascular ultrasound, ruled out pulmonary embolism, and routine laboratory results levels were within normal ranges. He was initiated on amlodipine at a dose of 10 mg per day to manage his hypertension.

During the night, the patient’s mental status deteriorated, leading to lethargy, flaccidity, and poor responsiveness (including unresponsiveness to sternal rub), in addition to elevated vital signs (temperature 97.6°F, blood pressure 166/77 mm Hg, pulse rate 108 bpm, respiratory rate 19 bpm, glucose level 144 mg/dL). Subsequently, he was transferred to the medical floor due to suspicions of a seizure with Todd's paralysis.

While on the medical floor, he was primarily treated for altered mental status and tachycardia. Laboratory tests, including creatine kinase (CK) levels, electroencephalogram (EEG), and a CT scan of the head, did not reveal any underlying organic causes for his psychomotor state. Neurology consultation failed to identify an obvious neurological explanation for his condition. The psychiatry consult team, however, noted that the patient met the diagnostic criteria for catatonia as per DSM-5 (Diagnostic and Statistical Manual of Mental Disorders, Fifth Edition) guidelines, displaying features such as mutism, stupor, autonomic abnormalities, a fixed gaze, and withdrawal from food and fluid. His Bush-Francis Catatonia Rating Scale (BFCRS) [4] score was 12 (Table 1). Intravenous administration of 2 mg of lorazepam promptly alleviated his catatonic symptoms, resulting in a BFCRS score of 0 within a minute. Subsequently, reintroduction to clozapine was undertaken cautiously, with the dose being escalated gradually back to 100 mg twice daily in the following 10 days. His psychotic symptoms resolved with clozapine titration and he did not need any further oral lorazepam doses in the subsequent days till his discharge from the hospital.

**Table 1:** BFCRS score [4] BFCRS, Bush-Francis Catatonia Rating Scale

	BFCRS score
Excitement	0
Stupor	2
Mutism	3
Staring	2
Posturing	0
Grimacing	0
Echopraxia/echolalia	0
Stereotypy	0
Mannerisms	0
Verbigeration	0
Rigidity	0
Negativism	0
Waxy flexibility	0
Withdrawal	3
Impulsivity	0
Automatic obedience	0
Mitgehen	0
Gegenhalten	0
Ambitendency	0
Grasp reflex	0
Perseveration	0
Combativeness	0
Autonomic abnormality	2
Total	12

## Discussion

Clozapine exhibits limited dopamine D2 receptor antagonist activity but exerts substantial serotonergic and muscarinic blocking effects [1]. Additionally, it possesses relatively modest direct GABA_A_ agonist properties but exerts indirect agonistic influence on GABAergic interneurons through interactions with other receptors. The withdrawal effects associated with clozapine primarily stem from cholinergic rebound, serotonergic rebound, and GABA supersensitivity [1]. Notably, these indirect effects on GABAergic interneurons can lead to the emergence of catatonic symptoms during clozapine withdrawal [1].

Clozapine, through its serotonergic 5HT2A antagonism and 5HT1A partial agonism, promotes dopamine elevation in the prefrontal cortex. Prolonged usage of clozapine results in the downregulation of dopamine receptors. Consequently, discontinuation of clozapine can precipitate a hypodopaminergic state, potentially triggering catatonic symptoms [1]. These effects are further exacerbated when other antipsychotic agents with prominent dopamine D2 receptor antagonistic properties are administered to such patients [5]. This might explain the worsening of catatonia in our patient when risperidone was introduced.

Cholinergic rebound during clozapine withdrawal can manifest as autonomic disturbances such as hypotension and bradycardia, gastrointestinal symptoms such as nausea, vomiting, and diarrhea, excessive salivation (sialorrhea), profuse sweating (diaphoresis), confusion, and psychosis. These symptoms can complicate the differentiation between clozapine withdrawal and a psychotic relapse [6].

Recognizing catatonia in the context of clozapine withdrawal can be challenging, especially since second-generation antipsychotics, including clozapine, tend to induce fewer motor abnormalities due to their low D2 receptor antagonism. Muscular rigidity and fever are less commonly reported in cases of neuroleptic malignant syndrome (NMS) associated with clozapine [7]. In our case, the patient presented with flaccid muscle tone, which confounded the diagnosis of catatonia for many clinicians. NMS can be precipitated similarly due to sudden changes in dopamine levels in the brain, but NMS was not suspected here due to the absence of rigidity, fever, and lack of elevation of CK levels in this patient.

When patients exhibit both psychotic and catatonic symptoms, clinicians face a dilemma regarding whether to initiate antipsychotic treatment. There have been numerous reports indicating that antipsychotic medications can exacerbate catatonia or induce NMS [5].

In the context of clozapine withdrawal-associated catatonia, re-initiating clozapine has been proposed as the optimal approach, given that benzodiazepines may be less effective [1,8]. However, re-initiating clozapine may not be feasible in patients with poor oral intake (due to catatonia) or low blood pressure (resulting from poor oral intake or cholinergic rebound). In such cases, alternative strategies may be considered, such as judicious use of benzodiazepines to address catatonic symptoms initially or the application of electroconvulsive therapy (ECT) [1]. Other alternatives include the use of anticholinergics to manage cholinergic rebound [9] or cyproheptadine to address serotonergic rebound [10]. Combining loxapine with cyproheptadine has also been recommended as this combination mimics the clozapine mechanism of action [11].

## Conclusions

Recognizing catatonia in the context of clozapine withdrawal can be challenging due to its atypical presentation. Re-initiating clozapine has been proposed as a treatment approach, but alternative strategies such as benzodiazepines, ECT, anticholinergics, cyproheptadine, or a combination of loxapine and cyproheptadine may be considered in patients with contraindications for clozapine re-initiation. Clinicians should exercise caution when managing such cases to optimize patient outcomes.

## References

[1] Lander M, Bastiampillai T, Sareen J (2018). Review of withdrawal catatonia: what does this reveal about clozapine?. Transl Psychiatry.

[2] Basu A, Jagtiani A, Gupta R (2014). Catatonia in mixed alcohol and benzodiazepine withdrawal. J Pharmacol Pharmacother.

[3] Ahmed S, Chengappa KN, Naidu VR, Baker RW, Parepally H, Schooler NR (1998). Clozapine withdrawal-emergent dystonias and dyskinesias: a case series. J Clin Psychiatry.

[4] Bush G, Fink M, Petrides G, Dowling F, Francis A (1996). Catatonia. I. Rating scale and standardized examination. Acta Psychiatr Scand.

[5] Lee JW, Robertson S (1997). Clozapine withdrawal catatonia and neuroleptic malignant syndrome: a case report. Ann Clin Psychiatry.

[6] Shiovitz TM, Welke TL, Tigel PD (1996). Cholinergic rebound and rapid onset psychosis following abrupt clozapine withdrawal. Schizophr Bull.

[7] Karagianis JL, Phillips LC, Hogan KP, LeDrew KK (1999). Clozapine-associated neuroleptic malignant syndrome: two new cases and a review of the literature. Ann Pharmacother.

[8] Beach SR, Gomez-Bernal F, Huffman JC, Fricchione GL (2017). Alternative treatment strategies for catatonia: A systematic review. Gen Hosp Psychiatry.

[9] Seppälä N, Kovio C, Leinonen E (2005). Effect of anticholinergics in preventing acute deterioration in patients undergoing abrupt clozapine withdrawal. CNS Drugs.

[10] Zesiewicz TA, Borra S, Hauser RA (2002). Clozapine withdrawal symptoms in a Parkinson's disease patient. Mov Disord.

[11] Aboueid L, McCarthy RH (2016). Loxapine and cyproheptadine combined limit clozapine rebound psychosis and may also predict clozapine response. Case Rep Psychiatry.

